# The Modulation of Spatial Working Memory by Emotional Stickers and Facial Expressions

**DOI:** 10.3389/fpsyg.2019.03082

**Published:** 2020-01-23

**Authors:** Yueying Li, Shengnan Li, Yanna Ren, Jianxin Chen, Weiping Yang

**Affiliations:** ^1^Department of Psychology, Faculty of Education, Hubei University, Wuhan, China; ^2^Department of Psychology, College of Humanities and Management, Guizhou University of Traditional Chinese Medicine, Guiyang, China; ^3^Brain and Cognition Research Center (BCRC), Faculty of Education, Hubei University, Wuhan, China

**Keywords:** spatial working memory, emotion, attention, stickers, faces

## Abstract

This article aims to investigate the interaction effects of emotional valence (negative, positive) and stimulus type (sticker, face) on attention allocation and information retrieval in spatial working memory (WM). The difference in recognition of emotional faces and stickers was also further explored. Using a high-resolution event-related potential (ERP) technique, a time-locked delayed matching-to-sample task (DMST) was employed that allowed separate investigations of target, delay, and probe phases. Twenty-two subjects participated in our experiment. The results indicated that negative face can catch early attention in information encoding, which was indicated by the augmentation of the attention-related P200 amplitude. In the delay phase, the N170 component represents facial specificity and showed a negative bias against stickers. For information retrieval, the increase in the emotion-related late positive component (LPC) showed that positive emotion could damage spatial WM and consume more cognitive resources. Moreover, stickers have the ability to catch an individual’s attention throughout the whole course of spatial WM with larger amplitudes of the attention-related P200, the negative slow wave (NSW), and the LPC. These findings highlight the role of stickers in different phases of spatial WM and provide new viewpoints for WM research on mental patients.

## Introduction

Working memory (WM) refers to the system responsible for the manipulation of information and temporary storage in the range of seconds and is important for a range of complex cognitive activities. Baddeley and Hitch (1992) hypothesized the existence of three major components in the WM system: (i) a phonological loop in the verbal WM system; (ii) a visuospatial sketchpad for the initial registration of non-verbal material in the visuospatial WM system; and (iii) a central executive component to determine which information should be made available for cognitive processing. These researchers also proposed the episodic buffer (Baddeley, 2000), which is assumed to play an attention-demanding role in binding together information from different sources.

As has been proposed by the processing efficiency theory (Eysenck and Calvo, 1992), negative emotions can have an impact on the phonological loop and central executive components, then further influence the cognitive process. Studies have suggested that spatial WM is vulnerable to negative emotion (Lavric et al., 2003; Weiland-Fiedler et al., 2004). For example, Lavric et al. (2003) found that anxiety can impair the performance of spatial WM in the *n*-back test. With the development of positive psychology, attention has been focused on the function of not only negative emotion but also positive emotion in the course of spatial WM. Negative emotion and positive emotion were shown to modulate WM through distinctive neural circuits, in which negative emotion activated the right amygdala and positive emotion activated the substantia nigra (Osaka et al., 2013). There were evidences showing that positive mood can impair spatial WM and executive control (Storbeck and Maswood, 2016), which can be demonstrated by the increased level of brain activation in spatial WM tasks (Gray, 2001). Moreover, spatial WM and emotion can serve as variables to better understand the psychopathology of mental disorder. There was growing evidence that dysfunctional spatial WM abilities may be linked to functional deficits in psychiatric disorders (Purcell et al., 1998a, b; Wee et al., 2003, 2007). For patients of depression, altered prefrontal brain activity was found during spatial WM task (Schecklmann et al., 2011). For obsessive compulsive disorder (OCD) patients, they perform poorly on tasks than engaging spatial WM (Purcell et al., 1998a, b), and showed abnormal activity in the anterior cingulate cortex (Wee et al., 2003). For schizophrenia, relative to healthy subjects, aberrant physiologic activity in the dorsolateral prefrontal cortex (DLPFC) is detectable, which engaged in the maintenance of spatial information (Glahn, 2000). That is to say, the extent of WM impairment can be used as an indicator to mental illness. For emotional perception, the impaired function manifests in flat, blunted and inappropriate affect, and can be reflecting on in emotion processing deficits in identification, discrimination and recognition of emotional facial expressions (Mandal et al., 1998). For patients with depression, the adopt of maladaptive emotion regulation strategies led to deficits in cognitive control (Joormann and Gotlib, 2010). For patients with schizophrenia, the failure to activate limbic regions during emotional valence discrimination may explain emotion processing deficit (Gur et al., 2002).

In our daily life, facial expressions can serve as social communicative signals to allow us to better estimate a person’s motivational state (Xu et al., 2015). The N170 component, which is shown in the temporo-occipital areas approximately 170 ms after the onset of a face stimulus, has been shown to be sensitive to facial stimuli (Bentin et al., 1996) and emotional facial expression (Schupp et al., 2004). Evidence shows a substantially stronger N170 for faces than non-facial objects, such as printed words (Marina, 2008) and cars (Dering et al., 2009). This kind of facial specificity can be explained by the expertise theory (Diamond and Carey, 1986), which argues that, as they possess a high degree of familiarity to faces, people are experts in recognizing facial information, thus inducing a stronger N170 component to faces than other non-facial stimuli. However, do stimuli which share similar features to faces also induce weaker N170 components than facial stimuli? With the rise in social media use, chatting online offers more flexibility for people to exchange information and provides a new way to communicate no matter where the users may be, during which non-verbal elements such as facial expressions, eye contact, and body movements are deprived. In order to compensate the lack of face-to-face communication between online environment, the use of stickers have become a common part in various forms of instant messaging use. A sticker can be simply textual, pictorial, or a combination of both, and can be either static or animated. It contains pictorial representations and can not only express the author’s emotions, but also represent social situations (Lee et al., 2016) and enhance human interaction (Derks et al., 2008). In light of the facial specificity of the N170 component, we hypothesized that even though faces and these stickers share similar features, faces can still induce a stronger, face-specific N170 component than stickers.

Previous studies have indicated that faces are capable of reallocating attentional resources in spatial WM tasks (Moriya et al., 2014). However, the attentional capture mechanism of stickers has seldom been mentioned. Compared to faces, we find that most of the stickers are based on the faces of familiar celebrities modified by means of caricature and exaggeration to some extent, potentially strengthening the impact, comprehension and interpretation of information by emphasizing its intended positivity, negativity, or neutrality (Skovholt et al., 2014) and are abundant in social content (Chang and Lee, 2016). In online communication, stickers are always used as supplements to the text message and can express the underlying intention by enriching the information (Suvorov and Dolin, 2007). We hypothesize that stickers, which share similar characteristics to faces and sometimes act as our “facial expression” online, can also capture attention in spatial WM and may actually catch more attention than faces because participants may think further about the internal information they contain. For emotional perception, it is obvious that stickers share similar emotional features with faces and, to some extent, can even result in stronger emotional arousals than faces because of their exaggerated expressions and high popularity in online social networking. Previous studies have shown that stickers can be used to accurately classify the emotional content of text messages in many cases and are easier for conveying information than text imputing (Lee et al., 2016). How the emotional valence of stickers can influence spatial WM is one of the topics of our study. The negative emotional state may induce individuals to care more about anxiety responses unrelated to the current task, which would distract attention and consume the limited WM resources, resulting in prolonged response times (RTs) and low efficiency of cognition (Shackman et al., 2006). In light of the negative bias (Carretié et al., 2001), we further hypothesize that negative stickers have a stronger ability to capture attention than positive stickers. In this study, with the help of high temporal resolution event-related potential (ERP), we intended to explore the brain mechanisms of spatial WM under induced positive and negative emotion in faces and stickers at different phases of information encoding, storage and retrieval in healthy participants. A number of ERP studies (Josiassen et al., 1982; Mecklinger and Pfeifer, 1996; Hajcak et al., 2010; Leppänen et al., 2010) have shown the ability of the P200 component and negative slow wave (NSW) to reflect WM attentional allocation as well as emotional perception by late positive component (LPC). In information encoding, after the preliminary classification and evaluation of items such as letters or spatial locations, the amplitude of the P200 component can reflect the degree of representation for WM, which has a close connection to the allocation of early attentional resources (Josiassen et al., 1982). Through the process of information storage, information is stored over time and represented in memory. Participants were instructed to engage in a sustained retention-rehearsal effort during the interval. Mecklinger found that there exists a bilateral posterior parietal occipital NSW component, which can reflect the storage and rehearsal of WM as well as the allocation of attention (Mecklinger and Pfeifer, 1996) and was thought to index early input operations, long-duration elaborative processes, and a rehearsal loop (Ruchkin et al., 2016). In information retrieval, parietal LPC have been found to be sensitive to emotion regulation (Hajcak et al., 2010) and to be associated with enhanced emotional processing (Leppänen et al., 2010). The LPC can also indicate sustained attention (Hajcak et al., 2010) and motivational significance (Schupp et al., 2000) to salient stimuli. The results of an ERP indicated that large LPC amplitudes are observed for positive emotions (Bublatzky et al., 2014) and that LPC amplitudes are decreased to aversive stimuli when compared to natural emotional states (Thiruchselvam et al., 2011). Previous studies indicated that mental diseases are often accompanied by attention deficit and emotional disorders (Mandal et al., 1998; Brandt et al., 2014). On the basis of our study, further research could be done on mental patients.

Delayed matching-to-sample task (DMST), which serves as a typical paradigm for spatial WM studies, can divide brain processing procedures into three parts: information encoding in the target phase, information storage and rehearsal in the delay phase, and information retrieval in the probe phase (Li et al., 2005). With the DMST, distinct brain processing procedures can be studied in separate phases. The main goal of this study was to examine the effect of the emotional states elicited from stickers and faces on attentional allocation in separate phases of spatial WM tasks as well as the interference of the interaction between emotion and stimulus types with spatial WM retrieval.

## Materials and Methods

### Participants

Twenty-two participants (8 men and 14 women; aged 18–23 years; mean age = 21.5 years) took part in the experiment, all right-handed and with normal or corrected-to-normal vision and hearing capabilities with no history of illness affecting brain function or a history of major psychiatric illness in first-degree relatives. The sample size of electroencephalogram (EEG) experiment was calculated by G-power on the basis of relevant studies. The participants provided written informed consent to participate in this study, which was previously approved by the Ethics Committee of Hubei University. All participants received payment for their time.

### Stimuli

For visual emotional stimuli, 64 facial affective stimuli pictures were selected from the Chinese Facial Emotional Picture System (Xu et al., 2011) and 64 affective sticker stimuli pictures were selected from WeChat, which is currently the most popular mobile instant messaging platform in China (Che and Cao, 2014). For each type of stimulus, 32 were classified as expressing positive (e.g., happiness) emotion and 32 were classified as expressing negative (e.g., anger, disgust, sad) emotions. All facial pictures were black and white (173 × 200 pixels, 24 bits). All sticker images were processed into 200 pixel width, black and white and without special body movement information using Adobe Photoshop. To pair the emotional valance of facial affective stimuli and affective sticker stimuli separately, an evaluation test was carried out. Sixty participants (27 male, 33 female, 19–22 years of age) were instructed to make an online evaluation on the emotional valence of the stimuli using a seven-point scale, where a value of 1 represented a very negative emotion, a value of 4 represented a neutral emotion, and a value of 7 represented a very positive emotion. A single sample *t*-test was conducted to eliminate data with no significant difference from the value of 4; pictures with high (value > 6) and low (value < 2) emotional valances were also removed. One positive facial stimulus (*p* < 0.05), one negative facial stimulus (*p* < 0.05), six positive Chinese sticker stimuli (*p* < 0.05) and four negative Chinese sticker stimuli (*p* < 0.05) were eliminated. The valence scores between positive and negative facial stimuli were significantly different (3.19 versus 5.19, *p* < 0.05) as were those for the sticker stimuli (2.58 versus 5.56, *p* < 0.05). Four types of stimuli (positive face, negative face, positive sticker, negative sticker) were paired separately with their equivalent emotional valances. For each type of stimulus, 26 pairs of images were produced to appear before and after the presentation of spatial WM tasks. The spatial WM task was a gray square, which was divided into nine equal pieces, four of which were randomly filled with black.

### Procedure

The experiment was conducted in a quiet and bright room (Laboratory Room; Hubei University, China). All stimuli were generated and displayed with E-prime 2.0 and presented on a monitor (1680 × 1050 pixels; 60 Hz). Participants sat 70 cm from the screen and were asked to remember the location of the black squares in the target phase and to judge whether the location of the black square that appeared in the probe phase was congruent with the location of one of the four black squares in the target phase. When the emotional faces or stickers were presented, participants were asked to identify the type of emotion and perceive the expressed emotion just mentally without making any key response to insure the processing of the type of emotion. Then, after seeing both of the emotional stimuli, they were required to press the left mouse button if the two were congruent or the right mouse button if the two were incongruent as fast and accurately as possible. All visual stimuli were presented with a visual signal of 10.7 × 8.7° and were at a visual angle of 3.4° to the left or right of center.

**FIGURE 1:**
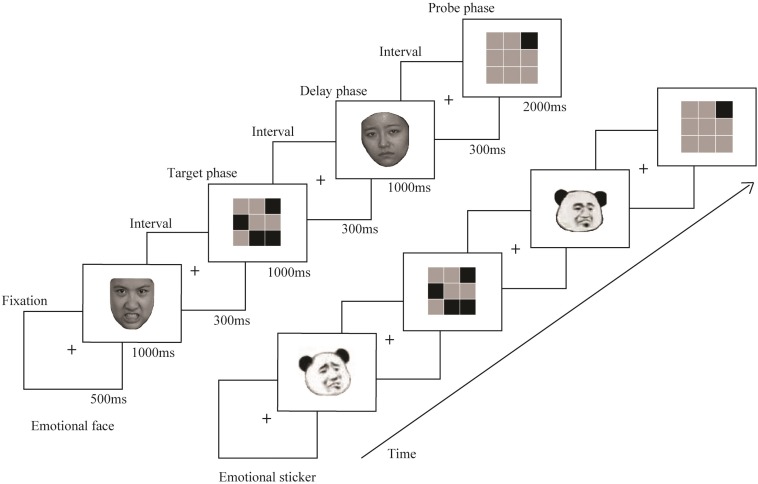
Design of the experimental paradigm.

Figure 1 shows the design of the experimental paradigm. At the beginning of each trial, a black fixation point appeared on a white background screen for 500 ms, and then an emotional picture (positive face, negative face, positive sticker or negative sticker) was presented on the screen for 1000 ms, followed by an interval of 300 ms. Then, the spatial WM task appeared for 1000 ms. After an interval of 300 ms, an emotional picture that was of the same stimulus type and emotional valance as the picture before the task was presented for 1000 ms in the delay phase. Finally, the probe stimulus was presented after an interval of 300 ms. A maximum of 2000 ms was available for responding; after that time, the next trial started. There were 4 blocks that each consisted of 52 trials. The four kinds of stimuli were presented at random to each participant in all blocks. Prior to the formal experiment, a practice experiment was conducted to ensure that the participants were familiar with the procedures.

### Apparatus

The EEG and behavioral data were recorded simultaneously. Stimulus presentation was controlled using E-prime 2.0. An EEG system (BrainAmp plus, Gilching, Germany) was used to record EEG signals through 32 electrodes mounted on an electrode cap (Easy-cap, Herrsching–Breitbrunn, Germany). All signals were referenced to FCz. Vertical eye movements and eye blinks were detected by deriving an electrooculogram (EOG) from a recording electrode positioned approximately one centimeter below the participant’s right eye, and horizontal eye movements were measured by deriving the EOG from one electrode placed at the outer canthi of the left eye. All electrode impedances were maintained below 5 kΩ. All electrodes were referenced offline to the average of both mastoids. The EEG and EOG were sampled at a digitization rate of 500 Hz.

### Data Analysis

#### Behavioral Data Analysis

The mean RTs were calculated based on the responses that fell within the average time period ±3 SD. The accuracy was the percentage of correct responses relative to the total number of target stimuli. The behavioral results for RTs and accuracy were analyzed using a 2 × 2 repeated-measures analysis of variance (ANOVA, Greenhouse–Geisser corrections with corrected degrees of freedom) with Emotion (negative/positive) and Stimulus type (face/sticker) as within-subject factors. The statistical significance level was set at *p* < 0.05 (Mauchly’s sphericity test). The effect size estimates η_p_^2^ were reported.

#### ERP Data Analysis

Electroencephalogram data were analyzed using Brain Vision Analyzer software (Version 2.0, Brain Products GmbH, Munich, Bavaria, Germany). ERP data were analyzed offline for only those trials on which the performance was correct. EEG and EOG signals were epoched into periods of 1150 ms, from 150 ms before the stimulus onset to 1000 ms after onset, and baseline corrections were made from −150 to 0 ms relative to stimulus onset. Trials with artifacts were rejected with a criterion of ±80 μV. These trials were subject to automatic rejection from the analysis. Then, the remaining trials were averaged separately for each participant, each session and each stimulus type following digital filtering using a bandpass filter of 0.05–30 Hz.

The following six sites were chosen for statistical analysis: F3, F4, and Fz (frontal) and P3, P4, and Pz (central-parietal). The amplitudes of the frontal P200 (the maximum positive peak in the time window 165–235 ms) for the target phase, the central-parietal N170 (the maximum negative peak in the time window 150–200 ms) and central-parietal NSW (the mean amplitude in the time window 450–850 ms) for the delay phase, and the frontal LPC (the mean amplitude in the time window 450–850 ms) for the probe phase were measured. Repeated-measures analysis of variance (ANOVA) was conducted on each ERP component with three factors: stimulus type (sticker/face), emotion (positive/negative), and electrode locations (F3, F4, Fz for P200 and LPC/P3, P4, Pz for N170 and NSW). Greenhouse–Geisser Epsilon correction was applied to adjust the degrees of freedom of the *F* ratios as necessary. All statistical analyses were carried out using SPSS version 16.0 software.

## Results

### Behavioral Results

The RTs and accuracy of each stimulus type are presented in Table 1. We performed 2 × 2 ANOVA with Emotion (negative/positive) and Stimulus type (face/sticker) as within-subject factors. For accuracy, the main effect of emotion [*F*_1_,_21_ = 7.180, *p* = 0.014, η_p_^2^ = 0.255] was significant, accuracy (positive) > accuracy (negative). The main effect of stimulus type [*F*_1_,_2__1_ = 0.041, *p* = 0.842, η_p_^2^ = 0.002] was not significant. There were no significant interactions between stimulus type and emotion [*F*_1_,_2__1_ = 0.388, *p* = 0.540, η_p_^2^ = 0.018]. For RTs, the main effect of stimulus type [*F*_1_,_21_ = 4.661, *p* = 0.043, η_p_^2^ = 0.182] was significant, RTs (face) > RTs (sticker). No main effect of emotion [*F*_1_,_2__1_ = 7.33, *p* = 0.402, η_p_^2^ = 0.034] and no significant interactions between stimulus type and emotion [*F*_1_,_2__1_ = 0.111, *p* = 0.742, η_p_^2^ = 0.005] were found.

**TABLE 1:** Mean and standard deviation of accuracy and response times.

**Stimulus type**	**Accuracy (%)**		**Response times (ms)**	
	**Positive**	**Negative**	**Positive**	**Negative**
Sticker	0.84 (0.12)	0.81 (0.09)	712 (171)	717 (146)
Face	0.83 (0.12)	0.80 (0.10)	732 (165)	745 (167)

*Standard deviations are given in parentheses.*

### ERP Results

**FIGURE 2:**
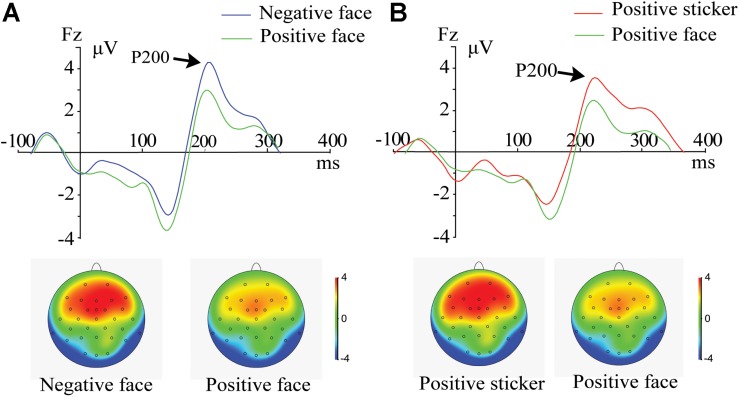
ERPs and amplitude topography of P200 (165–235 ms) for a representative channel (Fz) during the target phase. **(A)** P200 component for negative face versus positive face in the spatial working memory task. **(B)** P200 component for positive sticker versus positive face in the spatial working memory task.

For the target phase, a 2 (Emotion: positive, negative) × 2 (Stimulus type: face, sticker) × 3 (Electrode: F3, F4, Fz) ANOVA was performed. A significant main effect of stimulus type [*F*_1_,_21_ = 5.824, *p* = 0.025, η_p_^2^ = 0.217] was revealed for the frontal P200 amplitude. The amplitudes for trials following the presentation of stickers (3.66 ± 1.23 μV) were significantly stronger than those of the faces (3.02 ± 1.25 μV). There were significant interactions between stimulus type and emotion [*F*_1_,_21_ = 5.913, *p* = 0.024, η_p_^2^ = 0.220]. Further *post hoc* comparison results revealed that the P200 amplitude of the spatial task trials was stronger for the negative faces than for the positive faces (Figure 2A, *p* = 0.002) and stronger for the positive stickers than for the positive faces (Figure 2B, *p* = 0.008).

**FIGURE 3:**
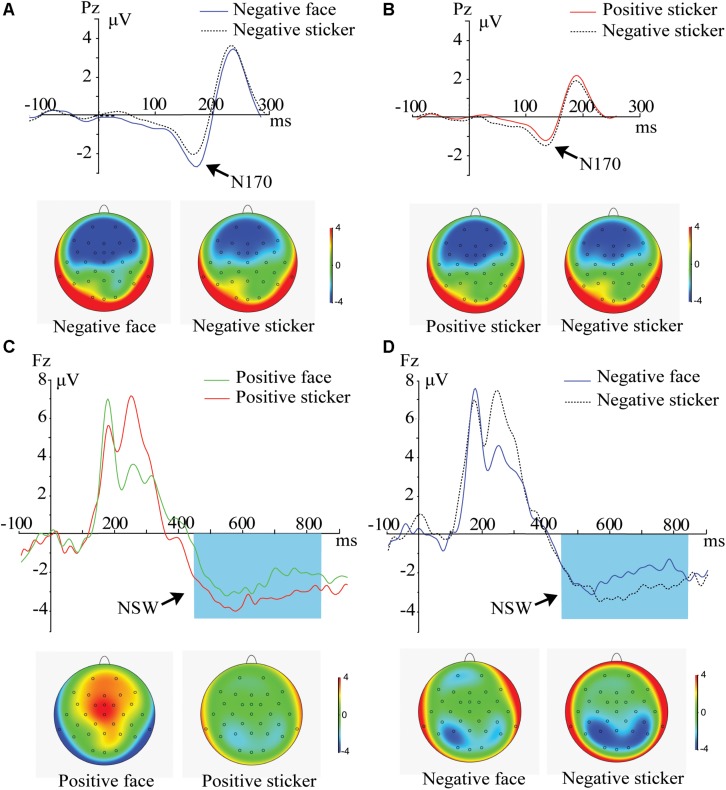
ERPs and amplitude topography of N170 (150–200 ms) and the NSW (450–850 ms) for the representative channel (Pz, Fz) during the delay phase. Blue boxes show the averaged time interval for the NSW component. **(A)** N170 component for negative face versus negative sticker in the spatial working memory task. **(B)** N170 component for positive sticker versus negative sticker in the spatial working memory task. **(C)** NSW component for positive face versus positive sticker in the spatial working memory task. **(D)** NSW component for negative face versus negative sticker in the spatial working memory task.

For delay phase, 2 (Emotion: positive, negative) × 2 (Stimulus type: face, sticker) × 3 (Electrode: P3, P4, Pz) ANOVA on central-parietal N170 and 2 (Emotion: positive, negative) × 2 (Stimulus type: face, sticker) x 3 (Electrode: P3, P4, Pz) ANOVA on central-parietal NSW components were performed. For the central-parietal N170 component, the main effect of electrode [*F*_2_,_42_ = 32.988, *p* < 0.001, η_p_^2^ = 0.767] was significant, and an emotion × stimulus type interaction was revealed [*F*_1_,_21_ = 5.608, *p* = 0.028, η_p_^2^ = 0.211]. *Post hoc* comparison results showed that in the negative emotion condition, the N170 amplitude was stronger for faces than stickers (Figure 3A, *p* = 0.008), and no difference was found for positive emotions (*p* = 0.775). In the sticker condition, the N170 amplitude was stronger for negative emotions than positive emotions (Figure 3B, *p* = 0.044), and no difference was found for the facial stimuli (*p* = 0.369).

For the central-parietal NSW component, the main effect of stimulus type [*F*_1_,_21_ = 11.521, *p* = 0.008, η_p_^2^ = 0.354] and electrode [*F*_2_,_42_ = 8.305, *p* < 0.004, η_p_^2^ = 0.454] was significant. The NSW amplitude for the stickers (−1.03 ± 0.29 μV) was significantly stronger than that of the faces (−0.52 ± 3.0 μV), as illustrated in Figures 3C,D. No significant interactions were found.

**FIGURE 4:**
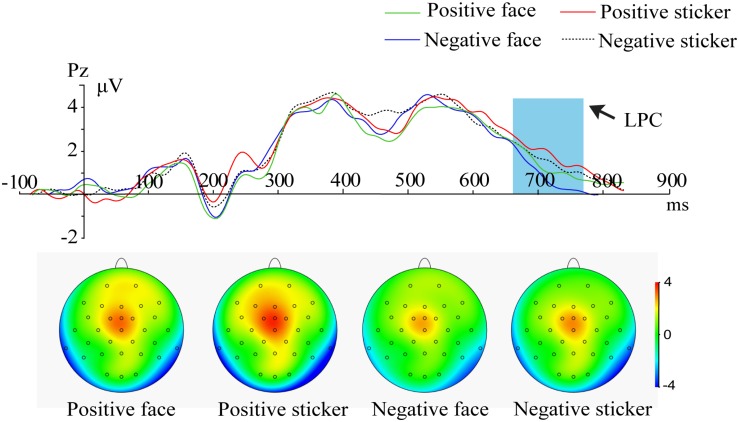
ERPs and amplitude topography of the LPC (660–760 ms) for the representative channel (Cz) in all conditions (positive face, negative face, positive sticker, negative sticker) during the probe phase. Blue boxes show the averaged time interval for the LPC.

For the probe phase, 2 (Emotion: positive, negative) × 2 (Stimulus type: face, sticker) × 3 (Electrode: F3, F4, Fz) ANOVA on the frontal LPC amplitude resulted in a significant main effect of stimulus type [*F*_1_,_21_ = 8.305, *p* = 0.003, η_p_^2^ = 0.454], and a larger amplitude of the LPC for stickers (2.66 ± 0.21 μV) than faces (2.42 ± 0.19 μV) was found. A main effect for emotion [*F*_2_,_42_ = 8.305, *p* = 0.005, η_p_^2^ = 0.454] was also significant; a stronger amplitude of the LPC for positive emotions (2.66 ± 0.21 μV) than negative emotions (2.42 ± 0.19 μV) was also indicated, as shown in Figure 4.

## Discussion

The main aim of the present study was to examine the interaction effect of emotion and stimulus type on attention allocation and information retrieval in spatial WM, as well as the difference in recognition for emotional faces and stickers. With the use of DMST, we assessed stimulus and emotional effects during the encoding, retention and retrieval periods of spatial WM.

In the target phase, facial stimuli with negative emotions can capture more early attentional resources in information encoding. Behavioral results demonstrated that negative emotion can lead to less accurate task performance. Previous studies have reported similar negative biases in the attention-related P200 component (Carretié et al., 2001). The results led the authors to conclude that facial stimuli with negative emotions are more capable of capturing attention (Huang and Luo, 2006). During spatial WM, spatial attention is in demand and may result in competition between cognition and emotion for attentional resources (Postle et al., 2004). As suggested in processing efficiency theory, the effects of negative emotion on cognitive processing may be mediated by the effects on WM (Eysenck and Calvo, 1992). The negative emotional state may induce individuals to care more about anxiety responses unrelated to the current task, which would distract attention and consume the limited WM resources, resulting in prolonged RTs and a low efficiency of cognition (Shackman et al., 2006).

For the delay phase, a stronger NSW was found for the stickers than for the faces during the interval. Evidence has shown that the NSW is related to the preparation for making the response required in the probe phase (Ruchkin et al., 2010), and so an increase in the NSW amplitude represents the increase of task difficulty (Mecklinger and Pfeifer, 1996). In the present study, more effort might be required when the task is followed by sticker than a facial stimulus during the interval. The possible explanation is that the presence of a sticker increased the task difficulty relative to that of the face stimulus condition, thus inducing larger retention-related NSW amplitude. When pictures were presented, participants were required to perceive the emotion. However, a sticker is not always a direct labeling of emotional content; it can sometimes be ambiguous (Koto and Adriani, 2015) and thus may add to the task difficulty.

For the probe phase, a positive sticker induced a stronger LPC than a negative sticker. The increase of the LPC amplitude reflects the effect of positive emotion on the processes of information retrieval in WM. As has been proved by studies on visual processing (Ostaszewski et al., 1998), negative emotions induced participants to focus on details, while positive emotions lead participants to fix on the overall structure and ignore the details, thus damaging detail-demanding spatial WM and consuming more cognitive resources. Our results can also be supported by ERP studies, which suggested larger LPC amplitudes for positive emotions (Bublatzky et al., 2014) and decreased LPC amplitudes to aversive stimuli when compared to neutral emotional states (Thiruchselvam et al., 2011). The statistical results of the N170 component indicated that, although sharing similar facial expressions to facial stimuli, negative stickers still induced a weaker N170 component than negative faces, which illustrated the specificity of facial recognition in N170. Previous studies have provided evidence for the face specificity of N170 (Cauquil et al., 2000) and typically illustrated a smaller or absent N170 response for non-face stimuli such as printed words (Carmel and Bentin, 2002) and cars (Dering et al., 2009), which reflects the absence of category-specific effects on N170 (Itier et al., 2006) and is in agreement with our findings. At least two processing steps are required for facial recognition (Sergent et al., 1994; Martin and Amanda, 2002). The first step is linked to the structural encoding of facial features, which occurs prior to facial identification and could be modulated by physical features. The perception of facial traits has been shown to consume more cognitive resources than stickers, which may reflect the face-specific processing mechanisms in the human brain (Mccarthy et al., 1997). The second step, which is thought to be the identification of emotional expression, is the result of the configurations of various facial features (Bruce and Young, 1986) and depends on the outflow of early raw information (Ashley and Vuilleumier, 2004). In our study, for sticker stimuli, negative emotions can induce a stronger N170 component than positive emotions, which can be interpreted by the unconscious mobilization of attentional bias toward negative information (Vuilleumier and Schwartz, 2015). Negative emotional states have long been held to serve as social contextual information to elicit attentional bias and narrow the scope of people’s attention and thinking (Murphy et al., 1999; Schmitz et al., 2009), in which anxiety is associated with the depletion of central executive resources and phonological resources, and can be associated with sub-vocal worry (Eysenck and Calvo, 1992). Some studies have proposed that the exogenous attention resources that have been automatically captured by negative emotions can no longer be used for other cognitive activities (Li et al., 2005), while others hold the view that visuospatial attention may be an overlapping area between negative emotion perception and spatial WM, which results in the impairment of spatial WM (Li et al., 2010a, b).

A notable finding of our study was the sustained larger attention-relevant P200 amplitude in the target phase, the stronger NSW component throughout the delay phase, and the larger LPC in the probe phase observed for stickers compared to facial stimuli. In light of this, the increased amplitude of P200 (Josiassen et al., 1982), NSW (Mecklinger and Pfeifer, 1996), and LPC (Hajcak et al., 2010; Weinberg and Hajcak, 2011) appear to have a connection with increased attention to stimuli. Moreover, the behavioral results indicated that sticker can lead to shorter RTs in task performance. Taken together, these results confirmed that stickers could capture attention throughout the entire spatial WM course. Stickers, which consist of abundant semantical and sentimental information, are widely used in daily communications to express people’s feelings and create a new form of language for social media users. Moreover, stickers can also demonstrate tone, intent and feelings that normally cannot be conveyed in digital messages and act as non-verbal cues in personal communications (Alshenqeeti, 2016); that is to say, stickers might capture more attention than faces. However, on account to the common existence of attention deficit among mentally ill people, does this kind of sustained attention to stickers still occur in mental patients is yet to be investigated. Moreover, stickers can serve as “emotion indicators” that embed rich, culturally relevant meanings and can integrate reality, social context and the virtual environment (Dresner and Herring, 2010). Stickers act not only as an emotional expression in mobile messaging but also as cues that include social content and personality (Chang and Lee, 2016). Relevant emotional recognition studies on mental patients can also be further facilitated.

In summary, the present study adopted DMST to focus on distinct brain processing procedures in information encoding, storage as well as retrieval separately, and provided some electrophysiological evidence for the interaction effects of emotional valence (negative, positive) and stimulus type (sticker, face) on attention allocation and information retrieval in spatial WM. We found that negative emotions can cause lower accuracy at the behavioral level. For information encoding, face with negative emotion can catch early attention. For information storage and rehearsal, the N170 component represents facial specificity and shows a negative bias against stickers. For information retrieval, positive emotions could damage in spatial WM and consume more cognitive resources. Moreover, stickers have the ability to catch attention on the entire course of spatial WM and lead to shorter RTs in task performance. However, several directions may be worth more research efforts. Firstly, in our study, emotional difference was only compared between negative and positive emotion for the value of amplitudes induced by certain ERP components. Further studies can add neutral emotion as baseline control condition to figure out whether there is any difference in quantity for interference or facilitating effect of emotion on spatial WM. Secondly, the sample size of this study was relatively small, which might cause failure to the discovery of interactions between emotional valence and stimulus type for NSW and LPC. Owing to this limitation, the results should to be considered preliminary, which need to be replicated in future studies with a larger sample size. Lastly, the interaction effects of emotional valence and stimulus type on attentional allocation and information retrieval in spatial WM was only studied on healthy participants preliminary. Previous studies indicated that mental diseases are often accompanied by attention deficit and emotional disorders (Mandal et al., 1998; Brandt et al., 2014). Whether the sustained attention to stickers and the impairment of positive emotion to spatial WM information retrieval that we found in our study occur to mental patients is yet to be investigated. On the basis of our study, future research can use psychopathology measures (e.g., anxiety, depression) to explore whether the sustained attention to sticker in spatial WM courses can still exist in mental patients.

## Data Availability Statement

The datasets generated for this study are available on request to the corresponding author.

## Ethics Statement

The studies involving human participants were reviewed and approved by the Ethics Committee of Hubei University. The patients/participants provided their written informed consent to participate in this study.

## Author Contributions

YL and WY wrote the manuscript. YL performed the experiments. SL analyzed the data. YR and WY conceived and designed the experiments. WY, SL, JC, and YR revised the manuscript and approved the final version.

## Conflict of Interest

The authors declare that the research was conducted in the absence of any commercial or financial relationships that could be construed as a potential conflict of interest.
